# Pain after Licorice or Sugar-Water Gargling in Patients Recovering from Oropharyngeal Surgery—A Randomized, Double-Blind Trial

**DOI:** 10.3390/jpm14101056

**Published:** 2024-10-12

**Authors:** Marita Windpassinger, Michal Prusak, Jana Gemeiner, Olga Plattner, Stefan Janik, Gerold Besser, Wolfgang Gstoettner, Pu Xuan, Daniel I. Sessler, Kurt Ruetzler

**Affiliations:** 1 Department of Anesthesia, Critical Care and Pain Medicine, Division of General Anesthesia and Intensive Care Medicine, Medical University Vienna, 1090 Vienna, Austria; 2 Outcomes Research Consortium, Houston, TX 77030, USA; ds@or.org (D.I.S.); kr@or.org (K.R.); 3 Department of Otorhinolaryngology, Medical University Vienna, 1090 Vienna, Austria; 4 Department of Quantitative Health Sciences, Cleveland Clinics, Cleveland, OH 44195, USA; 5 Department of Outcomes Research, Cleveland Clinics, Cleveland, OH 44195, USA; 6 Department of Anesthesiology and Center for Outcomes Research, University of Texas Health Science Center, Houston, TX 77030, USA; 7 Department of General Anesthesiology, Anesthesiology Institute, Cleveland Clinics, Cleveland, OH 44195, USA

**Keywords:** anesthesia, licorice, postoperative pain, analgesia, oropharyngeal surgery, gargling, pain management

## Abstract

Background: Glycyrrhiza glabrata (licorice) is used in traditional medicine and herbal remedies and reduces sore throats consequent to intubation, but whether it is protective for more intense pain after oropharyngeal surgery remains unclear. We thus tested the joint hypothesis that gargling with licorice, which has anti-inflammatory and antioxidant properties, reduces postoperative pain and morphine consumption. Methods: We enrolled patients having elective oropharyngeal surgery. Participants were randomly allocated to gargle with either 1 g licorice or a sugar placebo before and for up to three days after surgery. A numerical rating scale (NRS) for pain along with morphine consumption was evaluated every 30 min during the post-anesthesia care unit (PACU) stay and then three times daily for three days. We pre-specified that licorice gargling would be deemed better than sugar gargling only if found non-inferior on both morphine consumption and pain score and superior on at least one of the two. Results: 65 patients were randomized to the licorice group and 61 to placebo. We found noninferiority (NI) in pain scores with an estimated mean difference of −0.09 (95.2% CI: −0.88, 0.70; *p* = 0.001; NI delta = 1) between licorice and placebo gargling. There were no adverse events reported in either group that required treatment discontinuation. Conclusions: Gargling with licorice did not significantly or meaningfully reduce postoperative pain or morphine consumption in patients recovering from oropharyngeal surgery. While higher doses might prove more effective, our results suggest that other topical analgesics should be considered.

## 1. Introduction

Postoperative pain is a major cause of patient dissatisfaction [1]. Severe pain and difficulty in swallowing that compromise food intake are common in patients recovering from oropharyngeal surgery, and both impair well-being and sleep [2,3]. For example, Coulthard et al. reported that 93% of patients after oral and maxillofacial surgery experienced postoperative pain, ranging from moderate (47%) to severe (34%) [4]. Effective acute analgesia may prevent central sensation and the development of prolonged pain states. Pain is therefore a substantial issue for a large proportion of patients recovering from oral and maxillofacial surgery.

Topical treatments have the advantage of avoiding systemic effects, especially those caused by opioids. Various types of mouthwash and topical analgesic sprays reduce postoperative throat pain [5,6,7,8]. Gargling with an analgesic solution, for example, provides topical pain relief [9,10,11,12]. Postoperative licorice gargling is a well-documented method of preventing sore throats subsequent to intubation. Two trials each report that licorice reduces post-intubation sore throat, hoarseness, and coughing by a factor of two [13,14].

Licorice is a unique-tasting herb derived from glycyrrhiza glabra that contains various active ingredients, the most important of which are glycyrrhizic acid, glycyrrhizin, liquilitin, liquiritigeninglabridin, and hispaglabridins [15,16]. Glycyrrhizic acid is the main active ingredient in licorice root, and it is a triterpenoid saponin. It is a natural sweetener and flavoring agent commonly used in food and beverages, as well as in traditional medicine. Glycyrrhizic acid has various pharmacological effects, including anti-inflammation via inhibition of cyclooxygenase activity, prostaglandin formation, and platelet aggregation [17].

Glycyrrhetic acid is a metabolite of glycyrrhizic acid, formed by enzymatic cleavage of glycyrrhizic acid in the body. It is pharmacologically similar to glycyrrhizic acid and has similar anti-inflammatory, antioxidant and antiviral effects. Its other pharmacological activities include inhibiting type two 11-beta-hydroxysteroid dehydrogenase, which causes a cortisol-induced mineralocorticoid reaction [18]. Consequently, glycyrrhizin inhibits the production of pro-inflammatory cytokines and chemokines, which are involved in the initiation and progression of inflammatory processes [19]. Licorice flavonoids, such as liquiritigenin and isoliquiritigenin, also exhibit anti-inflammatory activity through the suppression of the nuclear factor kappa B pathway, which is involved in the regulation of immune responses and inflammation. Other anti-inflammatory properties include reduced expression of tumor necrosis factor-α, interleukin-6, inducible nitric oxidase synthase, and cyclooxygenase-2 in animal models [20,21].

A recent study evaluated the dose-dependence of licorice on postoperative sore throat, concluding that analgesia is better with 1 g licorice than at lower doses [22]. However, pain after oral surgery is far more intense than that from laryngoscopy and intubation. Whether gargling with licorice provides adequate analgesia after oropharyngeal surgery remains unknown. We therefore tested the joint primary hypotheses that gargling with 1 g licorice reduces pain scores and decreases morphine better than gargling with sugar water in patients recovering from elective oropharyngeal surgeries.

## 2. Materials and Methods

This trial was approved by the Medical University of Vienna Institutional Review Board (IRB #1308/2016), and written informed consent was obtained from all subjects participating in the trial. The trial was registered prior to patient enrollment at clinicaltrials.gov (NCT 02968823, Principal investigator: Olga Plattner MD, Date of registration: 16 November 2016).

### 2.1. Subject Selection

We enrolled children and adults between the ages of 12 and 99 years who were scheduled for elective oropharyngeal surgeries at the Department of Otorhinolaryngology in the Medical University Vienna, Austria. Legal guardians consented on behalf of patients <18 years old; patients 16–17 years old also assented; and patients ≥18 years old consented.

We included patients who had an American Society of Anesthesiologists (ASA) physical status 1–3. Elective oropharyngeal surgeries included panendoscopic surgery with larynx intubation using a small endotracheal tube followed by the introduction of a rigid airway for patients with tumors in the pharyngeal, hypopharyngeal, or laryngeal area. Other surgeries included tonsillectomy-adenotonsillectomies or biopsies for suspected tongue carcinoma.

We excluded patients with known or suspected allergies to licorice or its components, insulin-dependent diabetes mellitus, liver disease, or liver failure with bleeding disorders. We also excluded patients who took non-steroidal anti-inflammatory drugs within 24 h before surgery, chronically used morphine, were unable to use an intravenous patient-controlled analgesia pump, had a severely infected oropharyngeal tumor, required rapid sequence induction, or were expected to need mechanical ventilation after surgery.

### 2.2. Protocol

Participating patients were randomly assigned to gargle with licorice solution or placebo. Randomization was based on computer-generated codes with random blocking and no stratification. Assignments were kept in sequentially numbered opaque envelopes that were opened shortly before surgery. Allocation was thus concealed as long as practical.

The licorice solution consisted of 1 g licorice (extractum liquiritiae fluidum) diluted in 30 mL of water. The placebo solution was 5 g sugar, also diluted in 30 mL water, an amount that is comparably sweet. The solutions were kept in identical-looking bottles. Patients were asked to gargle with the trial solution for 60 s without swallowing, under the supervision and direct observation of an investigator not subsequently involved in data collection. Gargling was repeated two hours postoperatively and then three times daily for three days while patients remained hospitalized. As practical, gargling was timed to 30 min before eating. Patients, clinicians, and investigators were blinded to all treatments. Participating patients were told that two comparably sweet solutions were used for this trial but were not told what flavors were being tested or the trial hypothesis.

General anesthesia was induced with propofol (3–5 mg kg^−1^), fentanyl (3–5 µg kg^−1^) and rocuronium (0.6 mg kg^−1^) for muscle relaxation, and anesthesia was maintained with remifentanil 0.15–0.4 µg/kg/min and propofol 3–5 mg/kg/h. The trachea was gently intubated with a flexible tube size of 6–6.5 mm inner diameter for women and 7–7.5 mm inner diameter for men. Anesthetic administration was titrated to target a Narcotrend range between 40 and 55 (MonitorTechnik, Bad Bramdstedt, Germany) and to mean arterial pressure within 20% of pre-induction values. Mechanical ventilation with an oxygen-air mixture was adjusted to maintain end-tidal PCO_2_ between 32 and 35 mmHg as clinically practical.

Before transfer to the post-anesthesia care unit (PACU), patients were connected to an intravenous patient-controlled-analgesia (PCA) pump, starting with a bolus of 2 mg morphine hydrochloride at initiation of PCA, followed by demand doses of 2 mg with a lockout interval of 10 min and an hourly limit of 12 mg. A single dose of 500 mg metamizole IV (or paracetamol 500 mg in patients allergic to metamizole) was given during surgery. An additional dose was given in the PACU if necessary. Thereafter, pain was treated with morphine hydrochloride boluses per PCA until the first postoperative day, when patients were transitioned to oral metamizole, paracetamol, and dexibuprofen as necessary. Patients stayed at least one night in the hospital. They were discharged home with a supply of metamizole 500 mg tablets and dexibuprofen 400 mg tablets for analgesia.

### 2.3. Measurements

Demographic and morphometric characteristics, including sex, age, body weight, height, ASA Physical Status, and Mallampati Score were recorded. Anesthetic and surgical characteristics included type of surgery and total amounts of propofol, remifentanil, and fentanyl. Pain scores, incidence of coughing, and overall analgesic consumption during the initial three days after surgery were observed.

Patients were asked to record the severity of pain, cough intensity, adverse effects, and pain medications three times daily on each of the initial 3 postoperative days. Verbal pain scores were based on a Likert scale from 0 to 10, with 0 signifying no pain and 10 signifying the worst imaginable pain. Pain scores were recorded immediately after tracheal extubation every 30 min in the PACU for the first 2 h, on surgical wards 1 h after gargling, and then daily for up to 3 postoperative days at home. Other outcome assessments included coughing intensity (rated as none, mild, moderate, or severe) at 30, 60, 90, and 120 min after arrival in the PACU.

### 2.4. Statistical Methods

Demographic characteristics, including age, sex, weight, and race, ASA physical status, Mallampati score, surgery type, and intraoperative anesthetic variables, are summarized in Table 1. Baseline variables with an absolute standardized difference (ASD) > 0.349 (i.e., 1.96$\times \sqrt{\frac{1}{n_{1}}+\frac{1}{n_{2}}}$), where n_1_ and n_2_ are the number of patients in each group, were considered imbalanced and adjusted for in all analyses. Analyses were conducted based on a modified intent-to-treat basis where we included all randomized patients who received the treatment at least once.

#### 2.4.1. Primary Analysis

The primary outcomes were total morphine consumption and pain scores at rest during the first 2 h after the surgery. The effect of licorice gargling on pain scores and total morphine consumption during the first 2 h after arrival in PACU was assessed using a “joint hypothesis testing” framework. Using this framework, we pre-specified that licorice gargling would be deemed better than sugar gargling only if it was found to be non-inferior on both morphine consumption and pain score and superior on at least one of the two (Supplemental Figure S1).

We assessed the treatment effect on pain score over time in a repeated-measures linear model assuming autoregressive within-subject correlation structure. If there was a treatment-by-time interaction detected (*p* < 0.10), we would assess group differences at each measurement time. If the interaction was not significant, we would assess the group difference over all the measurements. We assessed the effect on morphine consumption in a linear regression model using log-transformed morphine consumption as the outcome, with the treatment effect expressed as a ratio of geometric means.

Using the estimated difference between licorice and sugar-water groups, noninferiority was tested using both the confidence interval method and a statistical test for noninferiority. Specifically, noninferiority would be claimed for morphine consumption if the upper limit of the 95% 2-sided confidence interval for the ratio of geometric means of total morphine consumption was less than the noninferiority delta of 1.15, and for pain if the difference in mean scores was less than the noninferiority delta of 1 (on our 11 point scale).

If noninferiority was claimed on both primary outcomes, superiority testing in the same direction would be conducted using the confidence interval method and statistical tests. Superiority would be claimed if the upper limit of the 97.5% confidence interval of the ratio of means was less than 1 for morphine consumption or if the 97.5% confidence interval of the pain score difference was less than 0 for pain scores.

#### 2.4.2. Secondary Analysis

We did not analyze the amount of coughing in the PACU since coughing scores were all 1 or 0; thus, an analysis of coughing incidence was sufficient. For pain, we used a generalized linear mixed model with binomial distribution with logit link assuming a covariance type of variance components (VC) to account for within-subject correlation. For pain scores from the end of surgery until the first postoperative morning, pain scores during postoperative day 1 (POD1) up to postoperative day 3 (POD3), separate linear mixed models were used to estimate the treatment effect on each corresponding outcome, assuming an autoregressive correlation structure to account for within-subject correlation. For total morphine consumption until postoperative day 1 morning and pain medication during POD1-POD3, a separate generalized linear model with Gaussian distribution and identity link was used with log-transformation applied on each corresponding cumulative morphine consumption.

The significance level for joint hypothesis testing was 0.05 overall and thus 0.025 overall in one direction for the noninferiority and superiority tests. No adjustment for 2 noninferiority tests was needed since noninferiority was required on both primary outcomes (95% CI used for each noninferiority test). Bonferroni correction was used for superiority testing since significance on either outcome was sufficient (97.5% confidence interval CI used for superiority tests). The significance level was 0.024 for this final analysis for noninferiority testing after adjustment for the sequential design, specifically spending alpha of 0.001 at the first interim analysis). Thus, 95.2% confidence intervals were calculated for the noninferiority tests. The significance level was 0.05 overall for the two-sided tests in secondary analyses, and thus the significance criterion was 0.01 for each secondary analysis after Bonferroni correction for multiple testing (0.05/5 = 0.01).

### 2.5. Sample Size and Power

A total of 252 patients were needed to achieve 90% power to detect noninferiority on both pain score (noninferiority delta of 1 on a scale of 0–10) and total morphine consumption (noninferiority delta of 1.15) and superiority on either outcome at the overall 0.025 significance level, including 3 interim analyses. Power was estimated using the statistical software suite (SAS) macro (developed for designs with joint hypothesis testing) and the SAS Seq Design procedure. The power calculation was based on 1000 simulations, assuming a mean (SD) pain score of 2 (2) and 3 (2) for the licorice and the sugar-water groups, respectively, and a coefficient of variation (standard deviation SD/mean) for morphine consumption of 0.25 for both groups. For both outcomes, we assumed an autoregressive correlation structure with an adjacent pairwise correlation of <0.30 and no interaction between group and time.

## 3. Results

**Figure 1:**
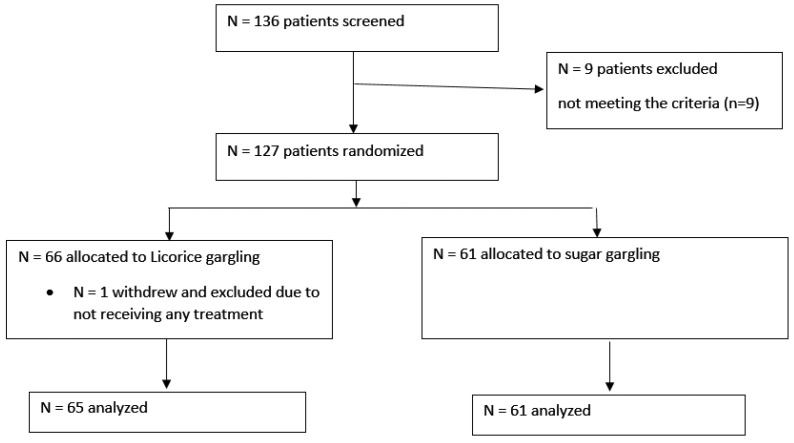
Patient Flowchart.

The Executive Committee stopped the trial after the second interim for pragmatic reasons related to enrollment difficulties. The decision was made without access to any by-group analyses. A total of 127 patients (licorice group *n* = 65, control group *n* = 61) were enrolled when the trial stopped, and 1 who received no trial treatment was excluded from the analysis (Figure 1).

The ASA physical status and type of surgery were unbalanced and thus were adjusted for analysis. The mean ± SD ages of licorice patients was 43 ± 19 years versus 37 ± 18 years in patients randomized to sugar water (Table 1). The mean NRS results for each group within different intervals after surgery are illustrated in Table 2.

We found noninferiority on pain score with an estimated difference in means of −0.09 (95.2% CI: −0.88, 0.70; *p* = 0.001; NI delta = 1) between licorice versus sugar gargling (Table 3). We did not find noninferiority on morphine consumption during the initial 2 postoperative hours, with an estimated ratio of geometric means of 0.85 (95.2% CI: 0.54, 1.36; *p* = 0.074; NI delta = 1.15) between licorice and sugar gargling. However, the absolute difference in median of morphine consumption was only 2 mg, which is not a clinically meaningful amount (Table 3). No superiority testing was conducted since we could not claim noninferiority on both primary outcomes.

We found a significant treatment-by-time interaction on the effect of licorice on coughing in PACU (*p* = 0.080). Thus, the treatment effect was estimated at each measurement time (Table 3). No significant treatment effect on coughing incidence was found at any measurement time (significance criterion = 0.0025 after multiple testing adjustment). Nor did we find any significant treatment effects for other secondary outcomes.

With the observed coefficient of variation (CV) of 0.85, we had a power of 0.18 to detect noninferiority with a noninferiority delta of 1.15 if we assume that the true ratio of geometric means is 1.0 between licorice and sugar gargling (Supplemental Figure S1) and a power of 0.78 if we assume the true ratio of geometric means is 0.8 at a significance level of 0.025 for one-sided test. The observed CV of 0.85 was higher than the 0.25 estimate used to estimate sample size, thus explaining our current actual power. Because it would have been impractical to enroll more patients, we did not conduct the planned re-estimation of the coefficient of variation.

This plot shows the power we had for detecting noninferiority on total morphine consumption with a noninferiority delta of 1.15 with different assumed treatment effects as ratios of geometric means, with observed coefficient variation of 0.85 and our current sample size of 126 at a significance level of 0.025.

## 4. Discussion

Previous work indicates that gargling with just 0.5 g of licorice reduces post-intubation pain and coughing [13,14]. Incisional pain is, of course, considerably more intense than post-intubation pain. We therefore used 1 g licorice dissolved in 30 g water. Nonetheless, gargling with licorice was significantly non-inferior on pain than gargling with sugar water. Morphine consumption during the initial 2 postoperative hours was not significantly non-inferior but differed by only 2 mg, which is not a clinically meaningful amount. Gargling with licorice thus provided little if any benefit on morphine consumption.

Topical analgesics and local anesthetics such as aspirin, diclofenac, ketamine, morphine, and lidocaine reportedly reduce postoperative pain, sore throat, discomfort, and even analgesic requirements within 24 h after surgery [5,6,7,8,9,10,11,12,23]. Gargling with a solution of honeysuckle and semen oroxyli (a traditional Chinese medicine) reportedly relieves both resting and swallowing throat pain during the initial 2 weeks after oropharyngeal surgery [24]. In contrast, various studies report that benzydamine hydrochloride, hydrogen peroxide oral rinses, or tantum verde mouthwash do not provide meaningful analgesia [25,26,27]. Two previous trials reported that gargling licorice before endotracheal intubation reduced postoperative sore throat and pain [13,14]. However, few trials directly compare the efficacy of various gargle solutions for analgesia after head and neck surgery. Our results indicate that licorice gargling provides trivial if any benefit after oropharyngeal surgery. Gargling licorice may thus reduce pain after minor airway trauma but is ineffective for more severe postsurgical pain.

Pain after head and neck surgery may partially result from colonization of bacteria in the surgical field and consequent painful inflammation—although the process presumably takes at least several hours. Oral rinses that reduce microbiological colonization and inflammation, such as hydrogen peroxide [27], benzydamine hydrochloride [26], and topical antibiotics [28], reduce postsurgical pain, presumably by reducing inflammation. A concern about perioperative licorice gargling is that the sweet herb might promote bacterial colonization within incisions, which might in turn augment pain. We saw no increase in pain over some hours, but longer-term effects remain possible. But given the lack of efficacy, it seems unlikely that licorice will be used for perioperative analgesia.

An obvious limitation of our trial is that it was stopped early for practical reasons related to low enrollment. However, there was no obvious benefit from licorice gargling, and it seems unlikely that a larger trial would demonstrate clinically meaningful benefit. Possibly a higher concentration of licorice, a different formula like gel or spray, and a combination with other anti-inflammatory and analgesic herbs would have been more effective. Sufficiently large amounts of ingested licorice can be toxic [29], but toxicity is not a concern when the herb is applied topically and not swallowed. Nonetheless, the dose is limited by the herb’s intense flavor, making higher concentrations increasingly unpalatable. Another limitation is our sugar placebo. Both solutions were comparably sweet, but based on its distinctive taste, patients would surely identify licorice. Without knowing the study hypothesis, it seems unlikely that identifying licorice would provide a substantial placebo effect—especially given the observed lack of benefit.

Patients were instructed to gargle three times a day for three days, which is consistent with previous studies, which specified treatments ranging from once daily [30] to every 6 h [24]. However, we failed to assess patient compliance or evaluate the adequacy of the gargling technique once patients left the post-anesthesia care unit. Poor compliance may therefore have compromised the treatment’s efficacy. Furthermore, the extent to which the intervals and duration of gargling influence analgesia remains unknown. Presumably more frequent and longer gargling would improve efficacy. It is also possible that an alternative application process would be more effective. For example, licorice lozenges would provide a longer exposure.

## 5. Conclusions

In summary, preinduction and postoperative licorice gargling did not produce meaningful analgesia or morphine sparing at any time during the initial three days after oropharyngeal surgery and is therefore discouraged in these patients. Our results contrast with previous trials reporting benefit from licorice gargling for less intense post-intubation sore throat. We conclude that clinicians consider alternative analgesic approaches for patients recovering from oropharyngeal surgery.

## Figures and Tables

**Table 1:** Baseline and demographic characteristics.

	Sugar (*n* = 61)	Licorice (*n* = 65)	ASD
Age, years	37 ± 18	43 ± 19	0.306
Female	25 (41)	31 (48)	0.135
White	60 (98)	65 (100)	0.183
Weight, kg	75 ± 17	74 ± 15	0.036
Height, cm	172 ± 9	171 ± 10	0.116
ASA status, *n*, %			0.468
1	41 (67)	29 (45)	
2	17 (28)	31 (48)	
3	3 (5)	5 (8)	
Mallampati score			0.345
1	40 (66)	35 (54)	
2	19 (31)	23 (35)	
3	2 (3)	6 (9)	
4	0 (0.0)	1 (2)	
Type of surgery			0.524
Pan-Endoscopy	17 (28)	30 (46)	
Tonsillectomy/Adenotonsillectomy	41 (67)	34 (52)	
Biopsy/demarcation of Ca of tongue	0 (0.0)	1 (2)	
Other	3 (5)	0 (0.0)	
Preoperative pain score	0.31 ± 0.87	0.37 ± 1.17	0.054
Intraoperative			
Propofol, mg	450 [360, 590]	385 [304, 515]	0.228
Remifentanil, mg	820 [461, 1000]	719 [500, 1000]	0.052
Fentanyl, mg	150 [100, 200]	150 [100, 200]	0.115
Metamizole, mg	1.00 [1.00, 1.00]	1.00 [1.00, 1.00]	0.008
Immediate cough after surgery			0.323
0	44 (72)	54 (83)	
1	13 (21)	7 (11)	
2	3 (5)	2 (3)	
3	1 (2)	2 (3)	

Variables are presented as means ± SDs, counts (percent), or medians [Q1, Q3] appropriately. ASA physical status: American Society of Anesthesiologists physical status. ASD: absolute standardized difference, defined as difference in means of proportions divided by pooled standard deviation. Baseline variables with absolute standardized difference (ASD) > 0.349 (i.e., 1.96$\times \sqrt{\frac{1}{n_{1}}+\frac{1}{n_{2}}}$) were considered imbalanced.

**Table 2:** The effect of licorice on secondary outcomes (*n* = 126) during PACU included 30, 60, 90, 120 min in PACU.

	Sugar (*n* = 61)	Licorice (*n* = 65)	Effect Estimate- Licorice Versus Sugar (99% CI) ^b^	*p* Value
During PACU				
Cough incidence			Odds ratio (99.75% CI) ^a^	
30 min	7 (11)	10 (15)	1.27 (0.25, 6.54)	0.656
60 min	11 (18)	3 (5)	0.19 (0.02, 1.51)	0.015
90 min	8 (13)	2 (3)	0.18 (0.02, 2.15)	0.036
120 min	6 (10)	4 (6)	0.53 (0.07, 4.16)	0.346
Until postoperative day 1 morning				
			Difference in means	
Pain score	2.8 ± 2.3	2.4 ± 2.4	−0.05 (−0.90, 0.79) ^c^	0.870
			Ratio of geometric means	
Total morphine consumption, mg	14 [4, 36]	8 [4, 31]	0.93 (0.60, 1.44) ^d^	0.665
POD1–POD3				
			Ratio of geometric means	
Total morphine consumption	12 [1, 25]	4 [0, 18]	0.80 (0.44, 1.47) ^d^	0.346
			Difference in means	
Pain score	2.3 ± 2.2	2.3 ± 2.5	0.14 (−0.73, 1.02) ^c^	0.668

Until postoperative day 1 morning included measurements in PACU and measurements afterwards until postoperative day 1 morning at 8 a.m. POD: postoperative day. Summary statistics are presented as *n* (%), means ± SDs, or medians [Q1, Q3]. The significance criteria was 0.01 for each secondary analysis after Bonferroni correction for multiple testing. ASA status and type of surgery were adjusted for in all the secondary analyses. (a) A generalized linear mixed model with binomial distribution with logit link assuming a covariance type of variance components (VC) to account for within-subject correlation was used. A significant treatment-by-time interaction was found on the effect of licorice on cough incidence during PACU (*p* = 0.080). The treatment effect was thus estimated at each separate measurement time along with 99.75% CI (0.01/4 = 0.0025) after multiple comparisons. (b) A 99% confidence interval was provided unless specified otherwise. (c) A linear mixed model assuming an autoregressive correlation structure for within subjects correlation was used to estimate the treatment effect on pain score. We did not find treatment-by-time interaction on either pain score until postoperative day 1 morning (*p* = 0.385) or during POD1-POD3 (*p* = 0.869). (d) A generalized linear model with Gaussian distribution and identity link with log transformed cumulative morphine consumption was fit to estimate the treatment effect on total morphine consumption.

**Table 3:** Primary noninferiority analysis: comparison on pain score and total morphine consumption during postoperative 2 h.

	Sugar (*n* = 61)	Licorice (*n* = 65)	NI Delta	Effect Estimate (95.2% CI), Noninferiority Test	Noninferiority *p*-Value ^§^
Pain score ^+^	2.9 ± 2.5	2.4 ± 2.5	1	Difference in means (Licorice versus sugar)	
				−0.09 (−0.88, 0.70)	0.001
Total morphine consumption (mg) ^‡^	6 [2, 11]	4 [2, 10]	1.15	Ratio of Geometric means (Licorice versus sugar)	
				0.85 (0.54, 1.36)	0.074

NI = noninferiority; CI = confidence interval. Summary statistics are presented as means ± SDs or medians [Q1, Q3]. ^§^ Significant when *p* < 0.024 for noninferiority test in this final analysis after adjusting *p* value for sequential design. Noninferiority would be claimed if the upper bound of CI does not exceed the NI delta (1 for pain score and 1.15 for total morphine consumption). ^+^ Difference in means of pain scores were assessed using a mixed-effects model with repeated measures with an auto-regressive correlation structure. The difference on pain score was not different over time (group-by-time interaction *p* = 0.759). ^‡^ Ratio of geometric means for morphine consumption between two groups was assessed using a linear regression model after logarithm transformation on total morphine consumption.

## Data Availability

The data presented in this study are available on request from the corresponding author.

## Supplementary Materials

The following supporting information can be downloaded at: https://www.mdpi.com/article/10.3390/jpm14101056/s1, Figure S1: Power to detect noninferiority on total morphine consumption in PACU assuming different treatment effect based on observed data.
