# Supplementary material for: Pain after Licorice or Sugar-Water Gargling in Patients Recovering from Oropharyngeal Surgery—A Randomized, Double-Blind Trial
Source: J Pers Med. 2024 Oct 12;14(10):1056. doi: 10.3390/jpm14101056 (PMC11508901; doi:10.3390/jpm14101056)
Supplement: Supplementary file 1 [file jpm-14-01056-s001.zip › jpm-3230693-supplementary.pdf]

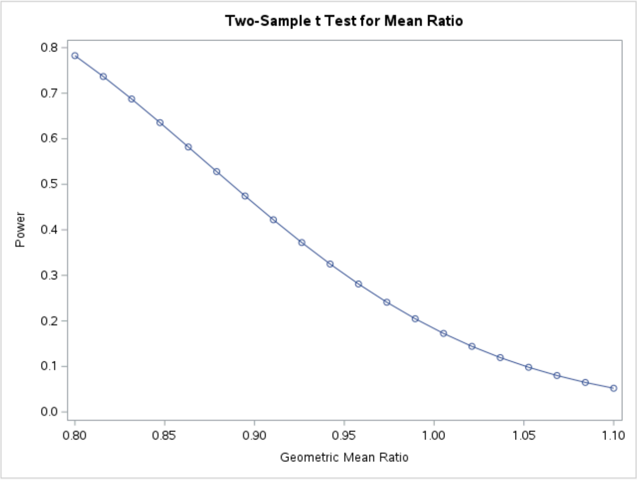

Supplemental Figure S1. Power to detect noninferiority on total morphine consumption in PACU assuming different treatment effect based on observed data.
